# Predictive value of the resistance of the probe to pass through the lesion in the diagnosis of peripheral pulmonary lesions using radial probe endobronchial ultrasound with a guide sheath

**DOI:** 10.3389/fonc.2023.1168870

**Published:** 2023-07-31

**Authors:** Zhenli Hu, Sen Tian, Xiangqi Wang, Qin Wang, Li Gao, Yuxuan Shi, Xiang Li, Yilian Tang, Wei Zhang, Yuchao Dong, Chong Bai, Haidong Huang

**Affiliations:** ^1^ Department of Respiratory and Critical Care Medicine, The First Affiliated Hospital of Naval Medical University, Shanghai, China; ^2^ Department of Respiratory and Critical Care Medicine, No. 906 Hospital of the Chinese People's Liberation Army Joint Logistic Support Force, Ningbo, China; ^3^ Department of Pathology, The First Affiliated Hospital of Naval Medical University, Shanghai, China; ^4^ Department of Nephrology, The First Affiliated Hospital of Naval Medical University, Shanghai, China; ^5^ Department of Respiratory and Critical Care Medicine, General Hospital of Central Theater Command of Chinese People’s Liberation Army, Wuhan, China; ^6^ Basic Medical School, Guizhou University of Traditional Chinese Medicine, Guiyang, Guizhou, China

**Keywords:** endobronchial ultrasound, transbronchial lung biopsy, peripheral pulmonary lesions, probe resistance, diagnosis

## Abstract

**Background:**

Transbronchial lung biopsy guided by radial probe endobronchial ultrasonography with a guide sheath (EBUS-GS-TBLB) is becoming a significant approach for diagnosing peripheral pulmonary lesions (PPLs). We aimed to explore the clinical value of the resistance of the probe to pass through the lesion in the diagnosis of PPLs when performing EBUS-GS-TBLB, and to determine the optimum number of EBUS-GS-TBLB.

**Methods:**

We performed a prospective, single-center study of 126 consecutive patients who underwent EBUS-GS-TBLB for solid and positive-bronchus-sign PPLs where the probe was located within the lesion from September 2019 to May 2022. The classification of probe resistance for each lesion was carried out by two bronchoscopists independently, and the final result depended on the bronchoscopist responsible for the procedures. The primary endpoint was the diagnostic yield according with the resistance pattern. The secondary endpoints were the optimum number of EBUS-GS-TBLB and factors affecting diagnostic yield. Procedural complications were also recorded.

**Results:**

The total diagnostic yield of EBUS-GS-TBLB was 77.8%, including 83.8% malignant and 67.4% benign diseases (P=0.033). Probe resistance type II displayed the highest diagnostic yield (87.5%), followed by type III (81.0%) and type I (61.1%). A significant difference between the diagnostic yield of malignant and benign diseases was detected in type II (P = 0.008), whereas others did not. Although most of the malignant PPLs with a definitive diagnosis using EBUS-GS-TBLB in type II or type III could be diagnosed in the first biopsy, the fourth biopsy contributed the most sufficient biopsy samples. In contrast, considerably limited tissue specimens could be obtained for each biopsy in type I. The inter-observer agreement of the two blinded bronchoscopists for the classification of probe resistance was excellent (κ = 0.84).

**Conclusion:**

The probe resistance is a useful predictive factor for successful EBUS-GS-TBLB diagnosis of solid and positive-bronchus-sign PPLs where the probe was located within the lesion. Four serial biopsies are appropriate for both probe resistance type II and type III, and additional diagnostic procedures are needed for type I.

## Introduction

There have been an increasing number of patients with lung nodules that are being detected owing to the increase of lung cancer screening by chest computed tomography (CT) scans, especially low-dose CT (1, 2). Of the nodules, more than 70% were peripheral pulmonary lesions (PPLs), according to the randomized NELSON trial (3). The rising number of PPLs has generated more urgent requirement of tissue verification to clarify a diagnosis. Despite the high diagnostic accuracy for CT-guided transthoracic needle biopsy (CT-TTNB) to identify PPLs (4), this technique is suboptimal as a result of radiation exposure and a considerable complication rate (5). By comparison with CT-TTNB, conventional flexible bronchoscopy is a minimally invasive procedure (6); however, its use in the diagnosis of PPLs remains a challenge with the differences in measures of precision and needle-to-target “miss” being major limitations (7). In view of this, radial probe endobronchial ultrasonography with a guide sheath (EBUS-GS) extending vision to PPLs was utilized to guide transbronchial lung biopsy (TBLB), with reported diagnostic yield of over 70% and lower complication rates (8, 9).

The factors affecting the diagnostic yield of EBUS-GS have been evaluated in a considerable amount of researches, most of which remains unclear with variations among studies (10–15); however, the position of the probe classified by the EBUS image is the most consistent and significant predictor associated with successful bronchoscopic diagnosis. The probe that is located within the PPLs provides the supreme diagnostic performance, followed by that located adjacent the PPLs and that located outside the PPLs (11–13, 16–19). In our clinical practice, in addition to the location relationship between probe and lesion, the propulsion resistance of the probe located within the lesion when passing through the lesion may be another underlying predictive factor in the diagnosis of PPLs. By far, to the best of our knowledge, there are still rare clinical reports concerning the corresponding relationship between different propulsion resistance of the probe and the PPLs diagnosis rate. Furthermore, as technology advances in the diagnosis and treatment (e.g. molecular targeted therapy) of lung cancer, adequate biopsy samples are warranted for pathological and molecular diagnosis. Nevertheless, currently, little if any research regarding the value of serial biopsies has been published.

Therefore, the present article was designed to confirm whether the probe resistance can influence the diagnostic yield of TBLB guided by EBUS-GS (EBUS-GS-TBLB) for PPLs. Also, we investigated the relationship between the yield of bronchoscopic biopsy specimens and serial biopsies, with the intention of presenting a strategy for sampling via EBUS-GS-TBLB in the clinical field, resolving the clinical contradiction corresponding to the yield of EBUS-GS-TBLB and prolongation of the intervention.

## Patients and methods

### Patient selection

We performed a prospective, single-center study investigation at a tertiary care academic medical center. All patients with PPLs who received EBUS-GS-TBLB were examined between 1 September 2019 and 31 May 2022 at the First Affiliated Hospital of Naval Medical University. Eligible and recruited patients must meet the following criteria: (1) the lesion is between 10 mm and 30 mm in diameter; (2) the nodule is solid; (3) the probe is located within the PPLs on a basis of the EBUS image; (4) CT scan shows the bronchus can be detected in connection with the lesion. Exclusion criteria: (1) the lesion can be seen directly under bronchoscopy; (2) fail to reach to the lesion using EBUS-GS; (3) absence of 1-year follow-up data after transbronchial procedure. The study protocol was approved by the Institutional Review Board of Shanghai Changhai Hospital, Naval Medical University (protocol code: CHCE2021-049). Written informed consent was obtained from all participants involved in the study.

### EBUS-GS-TBLB procedure

**Figure 1:**
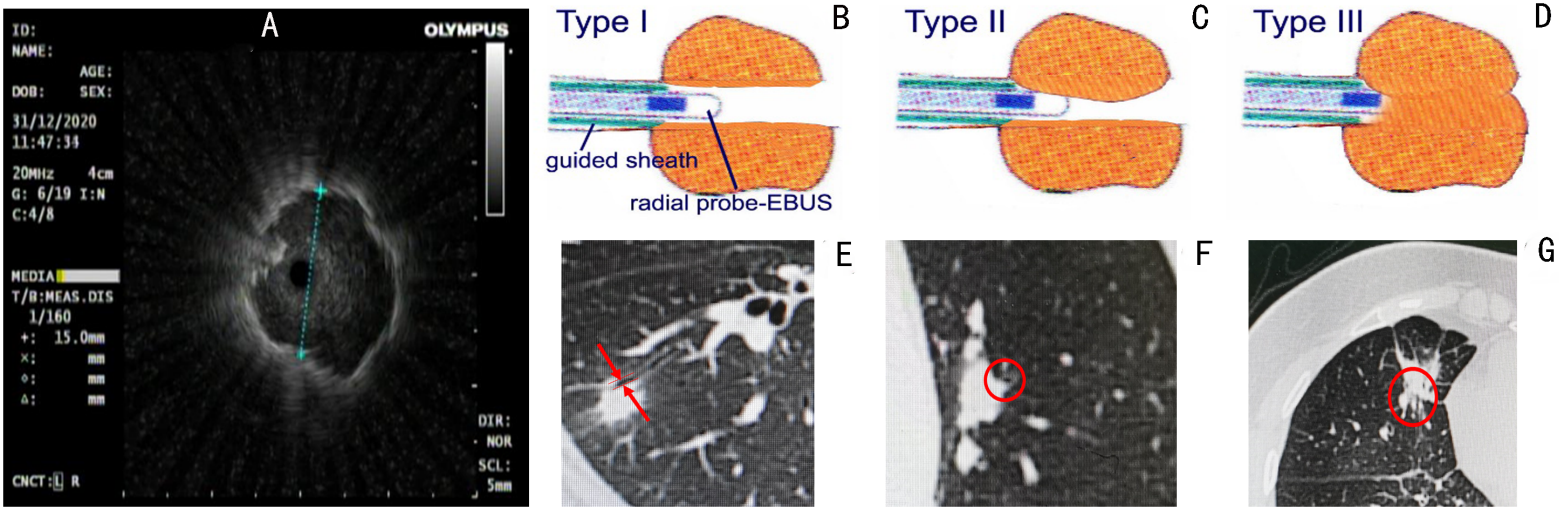
Radial probe endobronchial ultrasonography (EBUS) view of a peripheral target nodule where an EBUS probe is located within the target lesion **(A)**. We categorized the probe resistance in each case into three types I to III according to the different resistance of the probe to pass through the lesion. In type I, EBUS probe can pass through the lesion without resistance **(B)** where the bronchus in relation to the lesion was >1.4 mm in diameter **(E)**. In type II and type III, the corresponding bronchi were ≤ 1.4 mm in diameter **(F, G)**; for type II, EBUS probe could pass through the lesion with moderate resistance **(C)**, and for type III, EBUS probe could not pass through the lesion with high resistance **(D)**.

The size of the lesion measured on the axial lung window setting of chest CT, and the distribution of peripheral bronchus identified by 3 experienced bronchoscopists using chest CT, were utilized to determine the type of flexible bronchoscopes (BF260, BF P260F and BF P290, Olympus Corporation, Tokyo, Japan) prior to operation. All procedures throughout the study period were performed by the same interventional pulmonologist with more than 20 years of experience in the absence of any assistance from bronchoscopic navigation techniques. A 20-MHz radial-type EBUS probe with an external diameter of 1.4 mm (UM-S20-17S; Olympus, Tokyo, Japan) inserted through the working channel of the bronchoscope was applied in all patients. A guide sheath (GS) (SG-200C; Olympus, Tokyo, Japan) with an outer diameter of 1.95 mm was inserted together with the EBUS probe. Anesthesia was administered as previously described (14) and EBUS-GS-TBLB was conducted using 1.5-mm-diameter biopsy forceps (FB-233D; Olympus, Tokyo, Japan) according to the standard technology of Kurimoto et al. (16). When the probe located within the PPLs was advanced further (**Figure 1A**), we discovered the propulsion resistance of the probe. In each case, we categorized the probe resistance into three types (I to III). In type I, EBUS probe could pass through the lesion without resistance (**Figure 1B**) where the bronchus in relation to the lesion was > 1.4 mm in diameter (**Figure 1E**). In type II and type III, the corresponding bronchi were ≤ 1.4 mm in diameter (**Figures 1F, G**); for type II, EBUS probe could pass through the lesion with moderate resistance (**Figure 1C**), and for type III, EBUS probe could not pass through the lesion with high resistance (**Figure 1D**). The classification of probe resistance for each lesion was carried out by two bronchoscopists independently, and the final result depended on the bronchoscopist responsible for the procedure.

At least 6 successive biopsies were performed during EBUS-GS-TBLB, followed by bronchial brush cytology and bronchoalveolar lavage. Each biopsy was instantly presented onto a numbered glass slide and immediately evaluated by an experienced cytopathologist using rapid on-site evaluation (ROSE). Whether TBLB sampling ended was eventually determined by the bronchoscopist in the light of the feedback from ROSE.

### Diagnostic evaluation

The primary endpoint was the diagnostic yield according with the resistance pattern. The secondary endpoints were the optimum number of EBUS-GS-TBLB, factors affecting diagnostic yield, and incidence of procedural complications. In all cases, pathological examinations were performed by two experienced pathologists who analyzed the specimens obtained by EBUS-GS-TBLB. Diagnostic yield was calculated as the number of PPLs achieving successful diagnosis by EBUS-GS-TBLB divided by the total number of PPLs. The EBUS-GS-TBLB procedure was regarded as a definite diagnosis if the pathological assessment revealed malignant neoplasms, specific benign characteristics and/or positive microbiological results. For PPLs with nonspecific inflammation, only if the lesions decreased or disappeared were they considered to be successfully diagnosed by EBUS-GS-TBLB. For those non-diagnostic patients, additional diagnostic procedures, such as surgical biopsy and CT-TTNB, were required to further clarify. The final diagnoses were established on the basis of the pathological evidence, microbiologic evaluation, or clinical follow-up (20).

### Statistical analysis

The statistical analyses were carried out using SPSS Version 26.0 and GraphPad Prism Version 9.3.0. The continuous variables were presented as mean ± standard deviation (SD) unless otherwise stated. The Chi-square (χ2) test or Fisher’s exact test for the case of very small counts (≤5) was used to evaluate the differences in proportions. Every biopsy was reported and recorded separately with ROSE to build a quadratic function indicating incremental yield to plateau with serial biopsies and create a heatmap regarding to count of malignant cells for serial biopsies with different types of probe resistance. Inter-observer agreement was assessed using the weighted kappa (κ) coefficient. All analyses were two-tailed and a P value of <0.05 was considered significant.

## Results

### Patient characteristics

**Figure 2:**
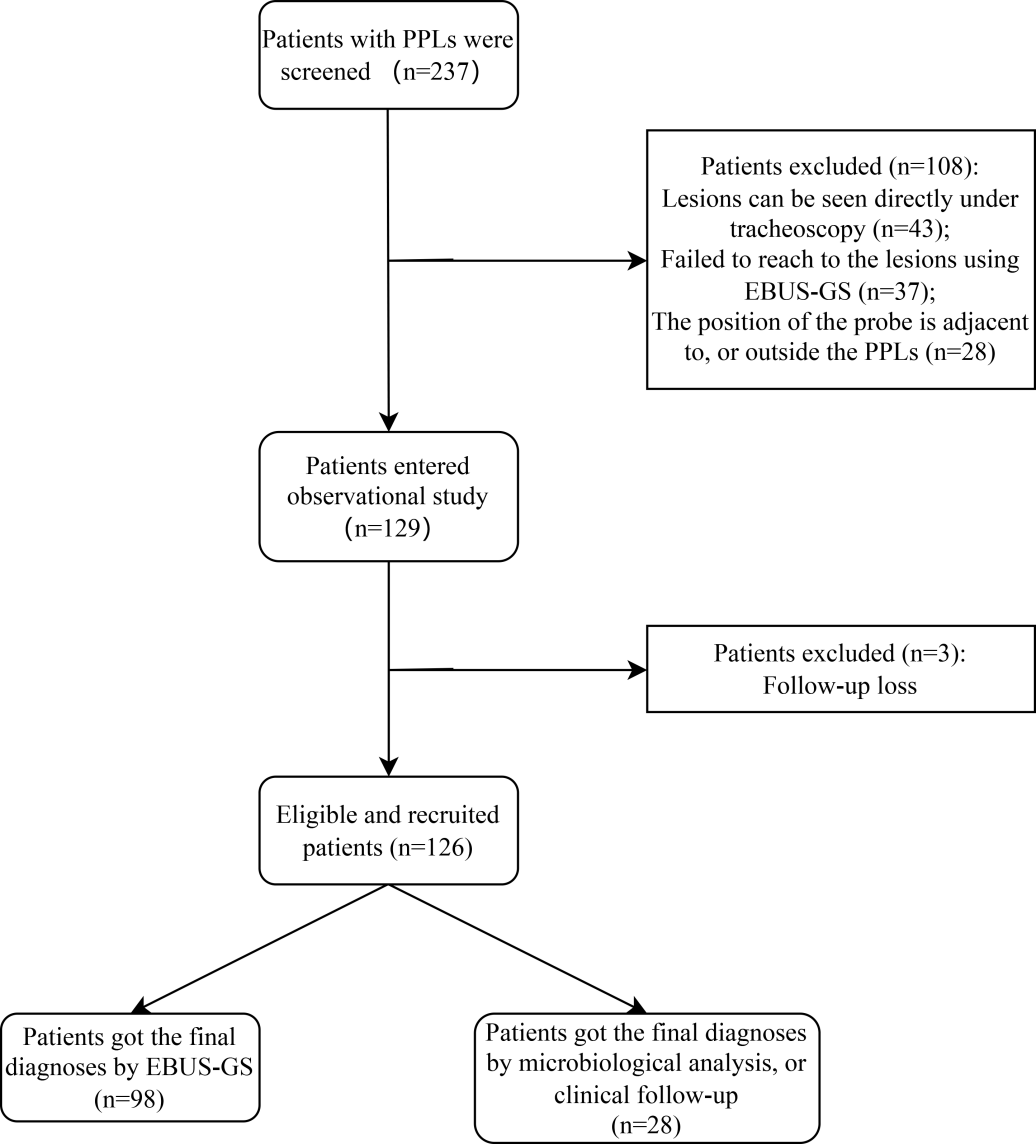
Flow diagram of study population selections. PPLs, peripheral pulmonary lesions; EBUS-GS, radial probe endobronchial ultrasonography with a guide sheath.

In total 237 consecutive patients with PPLs who underwent EBUS-GS-TBLB between September 2019 and May 2022 in our institution were obtained from the medical records and after screening, 126 eligible patients were ultimately included in our study (**Figure 2**). The basic characteristics of the studied patients are summarized in **Table 1** as follows. Of the 126 enrolled patients, the median age was 66 years (range: 45–79 years) and 60% were male. The mean size of the lesions was 19.7 mm (range: 10.0-30.0mm), and the localization of PPLs was the left upper segment in 31 (25%), lingula in 23 (18%), left lower lobe in 9 (7%), right upper lobe in 27 (21%), right middle lobe in 3 (2%) and left lower lobe in 33 (26%).

**Table 1:** Baseline characteristics of the study population.

Characteristics	n
**Age (Mean ± SD, years)**	64.98 ± 7.08
Sex	
Male	76 (60)
Female	50 (40)
**Size of lesion on CT (mean ± SD, cm)**	1.97 ± 0.81
Lobar location	
LUS	31 (25)
LLS	23 (18)
LLL	9 (7)
RUL	27 (21)
RML	3 (2)
RLL	33 (26)
Probe resistance^#^	
I	36 (29)
II	48 (38)
III	42 (33)

Data are presented as n (%) and %. ^#^see Patients and Methods.

CT, computed tomography; LUS, left upper segment; LLS, left lingular segment; LLL, left lower lobe; RUL, right upper lobe; RML, right middle lobe; RLL, right lower lobe.

The overall agreement of the two blinded bronchoscopists for the classification of the resistance of the probe to pass through the lesion was excellent (κ = 0.84, 95%CI: 0.76-0.93, P <0.001). Ultimately, we determined the type of probe resistance of every lesion: I (36 lesions, 29%), II (48 lesions, 38%), and III (42 lesions, 33%) (**Figures 1B–D**; **Table 1**). The final diagnosis of PPLs is presented in **Table 2**. Among them, 98 cases were diagnosed by EBUS-GS-TBLB, 10 cases by transbronchial cryobiopsy guided by EBUS (EBUS-TBCB), 6 cases by CT-TTNB, 4 cases by video-assisted thoracoscopic surgery (VATS), 4 cases by microbiological assessments, and 4 cases by clinical follow-up.

**Table 2:** Final diagnosis of PPLs (n = 126).

	Target sites biopsied
**Malignant**	80 (100)
Lung cancer	73 (91)
Adenocarcinoma	56 (77)
Squamous cell carcinoma	8 (11)
Small cell carcinoma	5 (7)
Other primary lung carcinoma	4 (5)
Metastatic	4 (5)
Lymphoma	3 (4)
**Benign**	46 (100)
Organizing pneumonia	13 (28)
Tuberculoma	10 (22)
Pulmonary aspergillosis	7 (15)
Bacterial pneumonia	7 (15)
Interstitial pneumonia	4 (9)
Sarcoidosis	3 (7)
Hamartoma	1 (2)
Pulmonary infarction	1 (2)

Data are presented as n (%) and %. PPLs, peripheral pulmonary lesions.

### Diagnostic yield of EBUS-GS-TBLB

In general, a definitive diagnosis of PPLs was obtained in 98 (77.8%) patients using EBUS-GS-TBLB and majority were adenocarcinoma. The diagnostic yield of malignant diseases was 83.8% (67/80), while benign diseases was 67.4% (31/46), with a significant difference (P = 0.033) (**Table 3**). Among the 15 patients with benign diseases not diagnosed by EBUS-GS-TBLB, pathological confirmation via EBUS-TBCB, VATS or CT-TTNB was achieved in 8, microbiological examinations in 4, and 3 underwent chest CT follow-up. We also analyzed the relationship between different propulsion resistance of the probe and the diagnostic rate. Type II exhibited the highest diagnostic yield (87.5%), followed by Type III (81.0%) and Type I (61.1%) (**Table 3**). Notably, of all 42 patients with a definitive diagnosis using EBUS-GS-TBLB in type II, 88.1% were malignant, which was significantly higher than benign diseases of 11.9% (P<0.001); in contrast, no significant changes emerged in the two other types.

**Table 3:** Diagnostic yield of all cases and different types of probe resistance.

		All cases	Probe resistance^#^		
			I	II	III
Diagnostic yield	All	77.8%(98/126)	61.1%(22/36)	87.5%(42/48)	81.0%(34/42)
	Malignant	83.8%(67/80)	66.7%(10/15)	94.9%(37/39)	83.3%(20/24)
	Benign	67.4%(31/46)	57.1%(12/21)	55.6%(5/9)	77.8%(14/18)
P value	intergroup*	0.033	0.732	0.008	0.955
	between-group^+^	–	0.005 0.393		
			0.052		

^#^See Patients and Methods; *Difference between malignant and benign lesions; ^+^Difference of all lesions with different types of probe resistance——The figures in top-hand part of the P value column show a comparison of probe resistance type I and II then type II and III; values in the bottom-hand P value column show a comparison of probe resistance type I and III.

For both size of lesion on CT and lobar location, no statistically significant differences in diagnostic yield were observed in any group. As shown in **Table 4**, pathologic characteristics of PPLs, malignant (10/17, 58.8% for type I; 20/24, 83.3% for type II) or benign (12/19, 63.2% for type I; 14/18, 77.8% for type II), did not influence the diagnostic yield of EBUS-GS-TBLB both type I (P=1.000) and type III (P=0.955). However, a significant difference between the diagnostic yield of malignant and benign diseases (37/39, 94.9% vs. 5/9, 55.6%) was detected in type II (P = 0.008).

**Table 4:** Comparison of diagnostic yield according to each parameter based on different types of probe resistance^#^.

Variables	I	P value	II	P value	III	P value
Size of lesion on CT		0.314		0.911		0.598
≤ 20mm	50.0%(7/14)		84.2%(16/19)		73.3%(11/15)	
> 20mm	68.2%(15/22)		89.7%(26/29)		85.2%(23/27)	
Lobar location		0.655		0.800		0.940
LUS and RUL	56.3%(9/16)		88.2%(15/17)		80.0%(20/25)	
LLS and RML	75.0%(6/8)		81.8%(9/11)		85.7%(6/7)	
LLL and RLL	58.3%(7/12)		90.0%(18/20)		80.0%(8/10)	
Lesion nature		1.000		0.008		0.955
Benign	63.2%(12/19)		55.6%(5/9)		77.8%(14/18)	
Malignant	58.8%(10/17)		94.9%(37/39)		83.3%(20/24)	
Total	61.1%(22/36)		87.5%(42/48)		81.0%(34/42)	

^#^See Patients and Methods. CT, computed tomography; LUS, left upper segment; RUL, right upper lobe; LLS, left lingular segment; RML, right middle lobe; LLL, left lower lobe; RLL, right lower lobe.

### Sequential yield of EBUS-GS-TBLB for malignant PPLs

**Figure 3:**
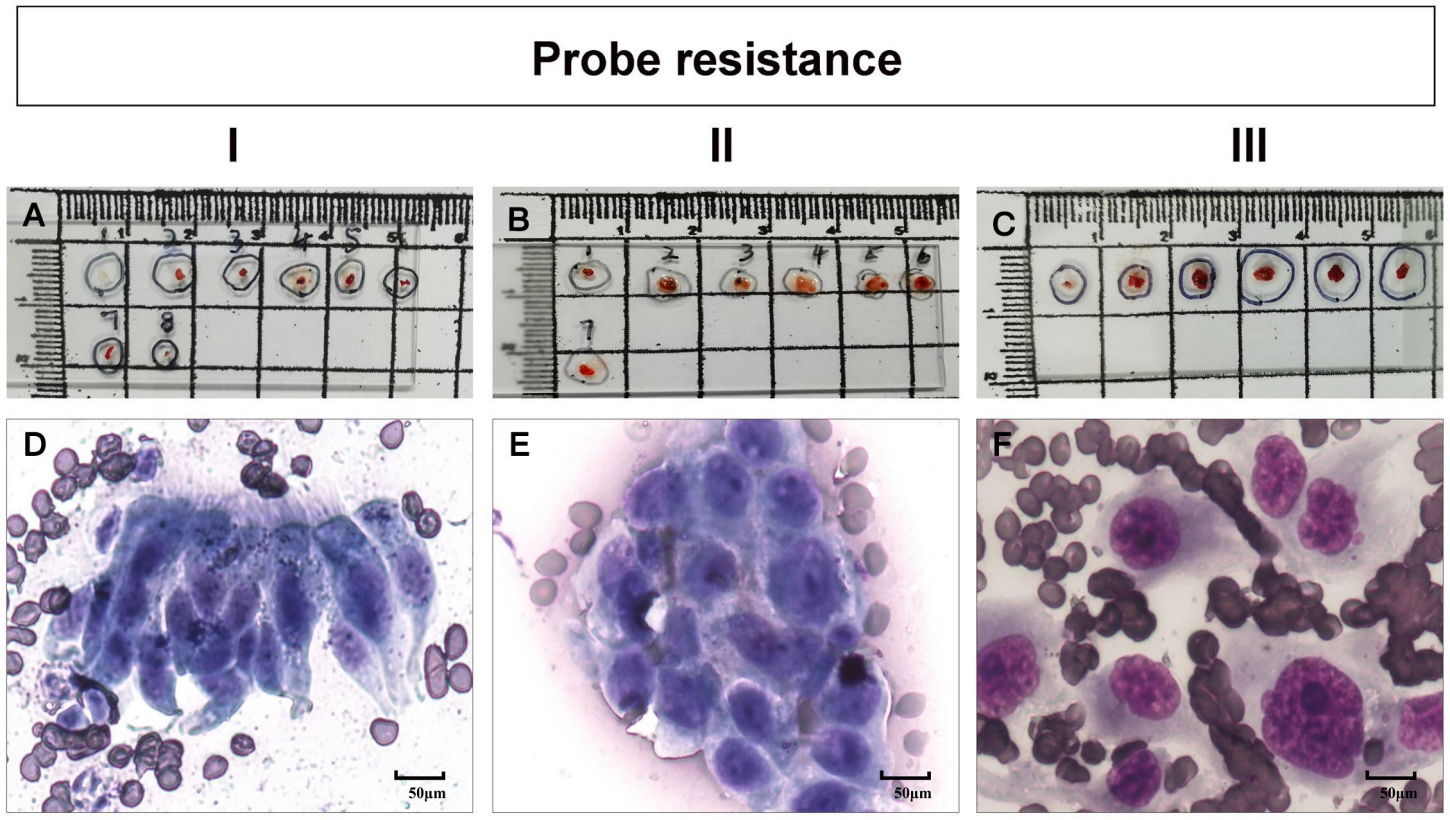
**(A–C)** bronchoscopic tissue specimen of different types of probe resistance (types I to III, see Patients and Methods), obtained by transbronchial lung biopsy guided by radial probe endobronchial ultrasonography with a guide sheath (EBUS-GS-TBLB). **(D–F)** corresponding cytology based on the rapid on-site evaluation (ROSE) revealing a cluster of ciliated columnar cells **(D)**, malignant cells **(E)** and several malignant cells, respectively.

**Figure 4:**
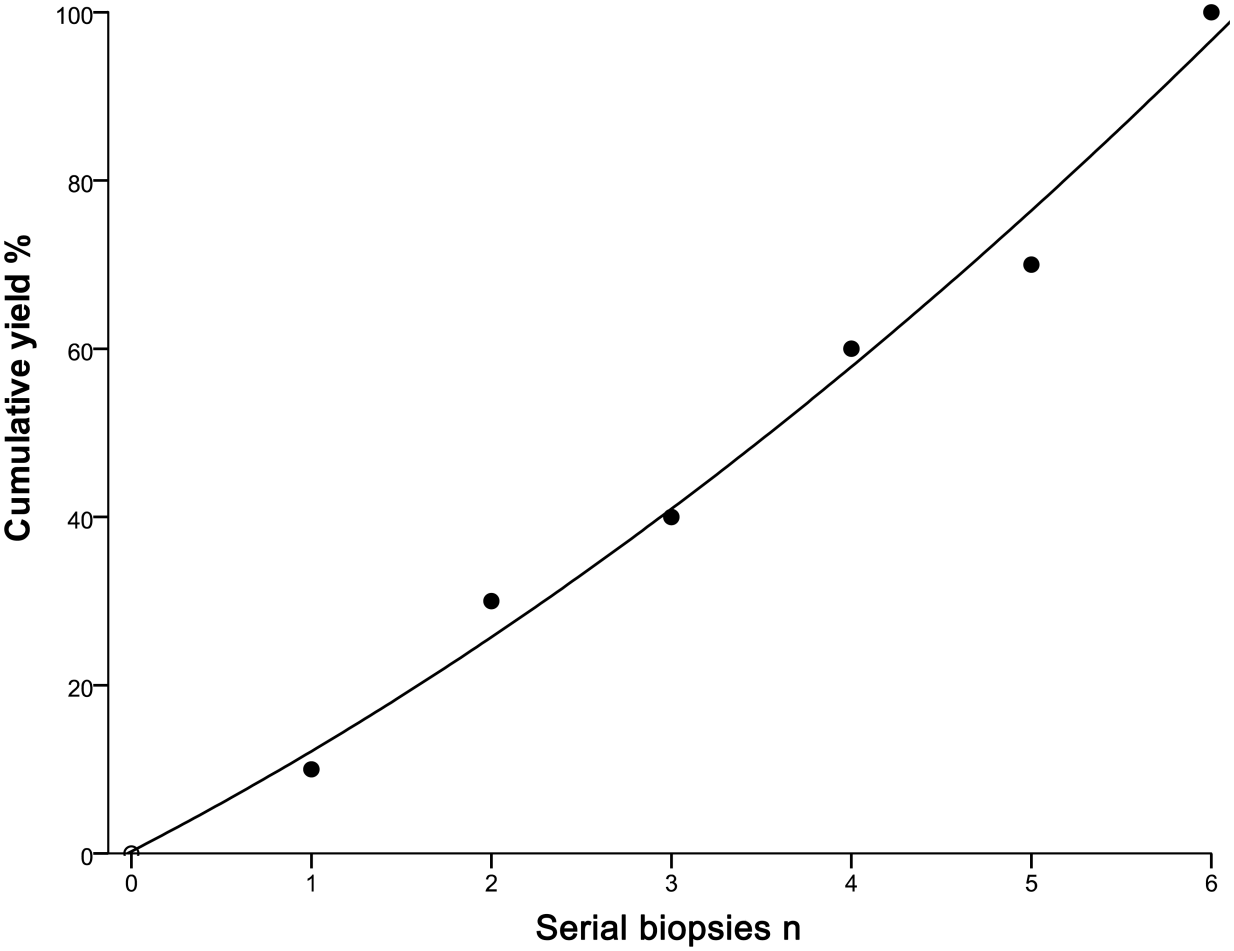
Incremental yield to plateau with serial biopsies. •: measured yield after each serial biopsy; —: extrapolated yield from a quadratic function, obtained by linear regression. The function is ‘‘yield = 0.24 + 11.07*serial biopsies +0.83*serial biopsies *serial biopsies’’, where the correlation is excellent (R^2^ = 0.989).

**Table 5** shows the rate of positive cells in the first biopsy for malignant PPLs with different types of probe resistance. The highest first-biopsy contribution was achieved at probe resistance with type III (100.0%), followed by type II (94.6%) and type I (10.0%). Corresponding cytology based on the ROSE revealed that malignant cells predominated in both type II (**Figure 3E**) and type III (**Figure 3F**), whereas ciliated columnar cells in type I (**Figure 3D**). For type I, it was observed that serial biopsies would result in incremental yield to plateau. As expected, a quadratic function obtained by linear regression could be employed to describe this pattern, with an excellent correlation (R^2^= 0.989) (**Figure 4**).

**Table 5:** Rate of positive cells in the first biopsy for malignant PPLs with different types of probe resistance.

	Probe resistance^#^		
	I	II	III
Rate of malignant cells*	10.0%(1/10)	94.6%(35/37)	100.0%(20/20)
P value^+^	<0.001 0.536		
	<0.001		

^#^See Patients and Methods; *based on the rapid on-site evaluation (ROSE) results; ^+^The figures in top-hand part of the P value column show a comparison of probe resistance type I and II then type II and III; values in the bottom-hand P value column show a comparison of probe resistance type I and III; PPLs, peripheral pulmonary lesions.

**Figure 5:**
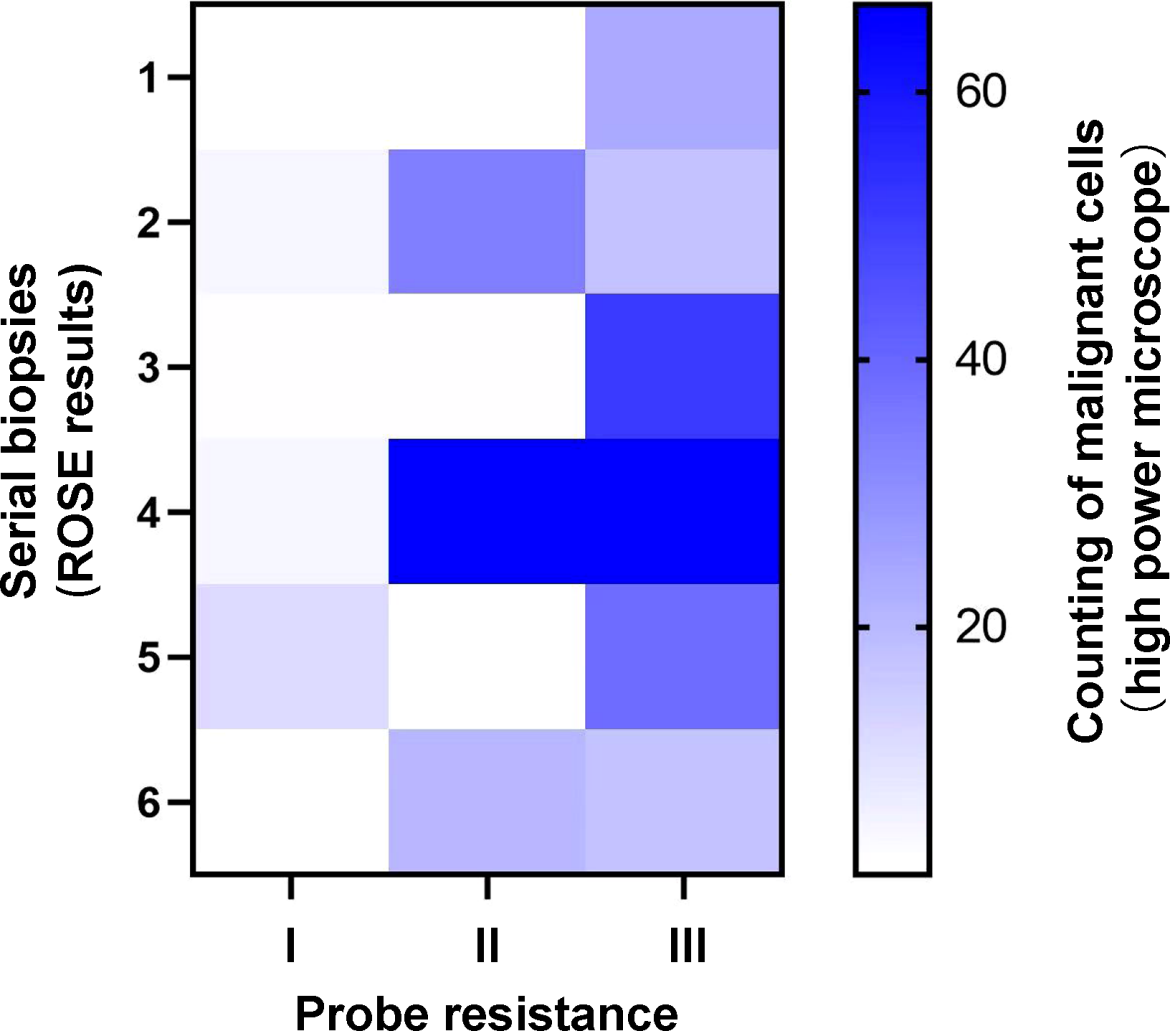
Heatmap of counting of malignant cells for serial biopsies with different types of probe resistance.

Intuitively, type II (**Figure 3B**) or type III (**Figure 3C**) could provide more bronchoscopic tissue specimens compared with type I (**Figure 3A**). To make it clear, we then investigated the amount of malignant cells for serial biopsies based on ROSE results in different types of probe resistance. As shown in **Figure 5**, although counting of malignant cells under the high power microscope was somewhat different for every biopsy in between type II and type III, the fourth biopsy consistently contributed the maximum amount of biopsy samples. By contrast, considerably limited tissue specimens could be obtained for every biopsy in type I.

### Complications

Moderate bleeding occurred in 4 (3.2%) cases that were controllable with topical application of cold saline solution with epinephrine. There were no pneumothorax or other severe complications associated with procedure occurring during the study period.

## Discussion

In the few decades, interventional pulmonology has evolved rapidly and revolutionized the analysis of PPLs as it enables the one-stop ambulatory bronchoscopic diagnosis and treatment of PPLs (21, 22); where EBUS-GS has undoubtedly contributed enormously to the recent advancement of interventional pulmonology (23). It is of key importance to determine whether EBUS-GS is suitable for patients with PPLs, and if appropriate, the optimal scheme of consecutive biopsies in order to avoid possibly unnecessary prolongation of the procedure remains unknown.

In the present study, the diagnostic yield of EBUS-GS-TBLB was 77.8%, which was in accordance with that of previous published reports (11, 14, 16, 24–27). In addition, a significant difference in the diagnostic yield was detected between malignant (83.8%) and benign (67.4%) diseases, demonstrating that the significance of EBUS-GS in the diagnosis of malignant PPLs was conspicuous and EBUS-GS was still necessary even for PPLs suspected to be benign (10). The complication rate with EBUS-GS-TBLB was only 3.2% from moderate bleeding, which served as the significant factor that this technique was recommended for PPLs diagnosis by the American College of Chest Physicians and European Society for Medical Oncology (28, 29).

Our results have indicated that the resistance of the probe to pass through the lesion is a significant predictive marker for successful EBUS-GS-TBLB diagnosis of PPLs. In detail, a probe resistance type II or type III is well correlated with the excellent diagnostic yield; for PPLs patients with which, EBUS-GS-TBLB examination is encouraged. On the contrary, an alternative or combined diagnostic procedure such as virtual bronchoscopic navigation (30) or electromagnetic navigation (31) should be considered if PPLs exhibits a probe resistance type I associated with the suboptimal diagnostic yield. Moreover, TBCB that can yield larger samples with better cellular architecture preservation and fewer crush artifacts seems helpful for improving the diagnostic yield of the probe resistance type I (32). Especially, in the present study, 9 PPLs patients with the probe resistance type I not diagnosed by EBUS-GS-TBLB underwent EBUS-TBCB and finally, definitive diagnoses were made in 7 cases with an acceptable safety profile.

Further analysis found this difference in diagnostic yield between malignant and benign PPLs is significant; the former is more likely to be successfully diagnosed than the latter. With respect to these observed results, possible reasons are listed as follows: malignant lesions invading the bronchial mucosa and resulting in propulsion resistance of the probe can be detected more easily. Moreover, when sampling for type III (the lesion in relation to high resistance), the forceps tips are often difficult to open the narrow lumen, thereby leading to its relatively inferior diagnostic yield in comparison with type II.

It is without dispute that this novel classification of contribution may provide useful reference for interventional pulmonologists when encountering PPLs to be determined. Surprisingly, the classification of probe resistance was identified with excellent inter-observer agreement. Whilst these are encouraging and promising, it is worthwhile noting that the classifications with regard to different types of probe resistance in all patients are assessed by the same bronchoscopist with more than 20 years of experience, and operator and expertise dependent factors in varying degrees may affect the classified types, as previously mentioned (33, 34). Consequently, further studies with a larger patient cohort are warranted to evaluate the inter-observer agreement and intra-observer reliability of this classification.

The ideal number of EBUS-GS-TBLB has not attracted considerable attention all the time, which may be attributed to high false negative rate of EBUS-GS-TBLB due to a variety of causes (e.g. unsatisfactory sample preparation and inadequate specimen collection) (35–37) and provision of the ROSE service allowing rapid stain and real-time assessment for direct slides to effectively optimize the number of biopsies and significantly increase diagnostic yield of EBUS-GS-TBLB (38–40). However, some also argue that ROSE is labor-intensive, does not improve PPLs diagnostics and makes it difficult to obtain biopsy samples available and sufficient for mutational analysis in case of need because of the waste of a considerable proportion of materials at the time of making direct slides (41–43). Plus, ROSE necessitating the presence of cytopathologists whose daily workload is too heavy to participate in this procedure is not universally available (39). In the absence of ROSE, it is common practice for the majority of interventional pulmonologists who are dependent on their clinical experience to decide on the number of biopsies, adding to uncertainty of the diagnostic yield and complication rates related to EBUS-GS-TBLB in the diagnosis of PPLs. When taking into account the above, we attempt to investigate the optimum number of EBUS-GS-TBLB. In our study, almost all of the malignant PPLs with a definitive diagnosis using EBUS-GS-TBLB in type II or type III could be diagnosed in the first biopsy; at the same time, the fourth biopsy contributed the most adequate specimens in these two types, which meant the decreased quantity of specimens available for diagnostic and subsequently molecular predictive evaluation even if more biopsies were performed. As such, it seems appropriate to conduct 4 successive biopsies for both probe resistance type II and type III so as to establish a histological diagnosis and perform molecular testing. Also, suboptimal amount of biopsy samples was noted in type I, suggesting that the addition of other available diagnostic procedures should be considered when the patients’ PPLs show the probe resistance type I.

This study carried certain limitations. First, this was an observational design with relatively small sample size in a single center. Because these results may vary among institutions, they should be interpreted with caution. Yet, as far as we are aware, this report is, for the first time, a tentative exploration on the usefulness of the probe resistance and the ideal number of EBUS-GS-TBLB. Hopefully, more multicenter prospective researches with larger patient cohort will be encouraged to have broader practice patterns, to reduce unnecessary bias and to improve the statistical power of our study. Second, the same bronchoscopist with more than 20 years of experience performed all procedures and clarified the probe resistance in our study; hence, universal adaptability of the clarification of the probe resistance remained to be established. Last, it was noted that the determination of the optimum number of EBUS-GS-TBLB was based on the ROSE examination; but ROSE could not take place of the final histopathologic evaluation. Furthermore, the evaluation concerning the sufficiency of EBUS-GS-TBLB specimens for molecular analysis was unavailable in the present study. Future studies are needed to be assured of the reliability of the conclusions.

Taken together, this observational study has demonstrated that the resistance of the probe to pass through the lesion has the potential to serve as a predictive factor for successful EBUS-GS-TBLB diagnosis of solid and positive-bronchus-sign PPLs where the probe was located within the lesion. Further, performing serial four biopsies is recommended for the probe resistance type II or type III, whereas additional diagnostic sampling methods are warranted for the probe resistance type I.

## Data availability statement

The original contributions presented in the study are included in the article/supplementary material. Further inquiries can be directed to the corresponding authors.

## Ethics statement

The studies involving human participants were reviewed and approved by the Institutional Review Board of Shanghai Changhai Hospital, Naval Medical University (protocol code: CHCE2021-049). The patients/participants provided their written informed consent to participate in this study.

## Author contributions

Conceptualization: ZH, ST, CB and HH. Methodology: ZH, ST, XW and QW. Investigation: XW, QW, LG, YT, WZ and YD. Analysis: ZH, ST, XW and XL. Funding acquisition: CB and HH. Project administration: WZ, YD and CB. Supervision: CB and HH. Writing original draft: ZH and ST. Writing-review and editing: CB and HH. All authors contributed to the article and approved the submitted version.
